# Family interaction among young Chinese breast cancer survivors

**DOI:** 10.1186/s12875-021-01476-y

**Published:** 2021-06-21

**Authors:** Jiehui Xu, Xiyi Wang, Mengjie Chen, Yiwen Shi, Yun Hu

**Affiliations:** 1 Department of Breast Surgery, Ren Ji Hospital, Shanghai Jiao Tong University School of Medicine, Shanghai, China; 2 School of Nursing, Shanghai Jiao Tong University, 227 South Chongqing road, Huangpu, Shanghai, 200025 China; 3 Xin Hua Hospital, Shanghai Jiao Tong University School of Medicine, Shanghai, China

**Keywords:** Young, Breast cancer survivors, Family, Interaction, China

## Abstract

**Background:**

Family interaction is an important factor contributing to the quality of survivorship among breast cancer survivors. The dearth of studies involving young females with breast cancer has limited the understanding of family interaction in this increasingly large population.

**Methods:**

The aim of this study was to explore family interaction patterns among young Chinese breast cancer survivors. We conducted in-depth interviews with seventeen young breast cancer survivors (YBCSs) in China between May 2019 and December 2019. A content analysis was performed to identify the characteristics of family interaction in this population. Conceptualizations of feminism and social support were used to guide the data analysis.

**Results:**

Family interaction patterns were categorized into 5 domains from the perceptions of Chinese YBCSs: (1) adjustment of parenthood (changes in child-rearing approaches, perception of children’s care) (2) ambivalence towards intimacy (desire for intimate relationships, perceived relationship insecurity); (3) concerns regarding fertility; (4) return to work (coping with gratitude and guilt by working, readapting to family and society by working); (5) activation of the support system in a large family (instrumental support from core family members, instrumental, informational, and appraisal support from relatives).

**Conclusions:**

The study provides a deeper understanding of the interactions between young breast cancer survivors and their family members in China. These findings can support health professionals in developing female-sensitive, culturally specific interventions to assist Chinese YBCSs and their families in increasing positive interactions and family resilience as well as quality of life. In addition, the findings are highly applicable to other female cancer survivors and their vulnerable families exposed to similar social and cultural contexts.

**Supplementary Information:**

The online version contains supplementary material available at 10.1186/s12875-021-01476-y.

## Background

Female breast cancer is the most commonly occurring cancer worldwide [1]. The estimated 2.3 million new cases in 2020 correspond to one in every 8 cancers diagnosed. In China, approximately 416,371 females were diagnosed with breast cancer in 2020, compared with 276,382 cases of lung cancer (the second most common cancer) in females [2]. Breast cancer rates and the number of young survivors are both increasing, and these trends are expected to persist for a long time [3–5]. Depending on race and the specific study, cut-off ages for survivors to be defined as young breast cancer survivors (YBCSs) of 35, 40, 45, and 50 have been applied [6–8]. In China, the first peak in the prevalence of breast cancer among Chinese women occurs at the age of 45, which is younger than that among Western women [9, 10]. Therefore, we recruited participants younger than 45 years of age for the present study.

YBCSs have unique clinical and biological characteristics and show poorer survival outcomes than female survivors of rectum cancer, cervix cancer, and melanoma [11]. In particular, the incidence of invasive breast cancer far exceeds the incidence of any other cancer among young women [12]. Young women with larger and/or more aggressive tumours are more likely to be treated with mastectomy, particularly contralateral prophylactic mastectomy. Biological and therapeutic factors contribute to the short-term and long-term health effects that affect the quality of life throughout YBCSs’ lifetimes [12].

Additionally, YBCSs face challenges and disruptions in their social roles as parents and income earners. Both YBCSs and their family members have been found to experience a poor quality of life [11, 13, 14]. Cohee [13] reported that lower marital satisfaction and greater hyperarousal were observed in families who faced the acute crisis of a breast cancer diagnosis. Thus, the activation of family resilience is critical for assisting YBCSs and increasing the quality of life of survivors and their families. Studies have increasingly reported that resilience during cancer diagnosis and treatment allows YBCSs to overcome daily stressors and reach a new balance in family functioning [15]. Furthermore, resilience was found to have a positive influence on the cancer trajectory [16].

Most studies addressing family resilience focused on the influence of well-functioning families on caregiver burden and quality of life among breast cancer survivors and their family members [15, 17, 18]. For example, Li et al. [15] reported that family resilience and breast cancer survivors’ individual resilience may ease the burden of primary family caregivers. Moreover, family resilience has been shown to promote survivors’ individual resilience in China. Another study examined the relationship between family well-being and the physical quality of life of breast cancer survivors in diverse ethnic groups [19]. However, far fewer studies examined the characteristics of family resilience related to cancer, especially among YBCSs. Family resilience can be assessed in detail at various levels. A family’s communication patterns and collective attribution style have been shown to reflect the quality of the family’s resilience at the microsystem level [20]. Adaptive and maladaptive interactions were found to coexist and be associated with positive and negative cycles of risk exposure [21]. Families who successfully adapted were reported to show a cohesive pattern of engagement and find a new family balance to effectively respond to the cancer crisis. During this response process, cultural factors contribute to the interaction between cancer survivors and family members during the experience of a cancer crisis. The young Chinese generation is subject to the complex influence of both their traditional culture and Western culture, thereby experiencing the conflicting values of individualism and collectivism and filial piety (*xiao*) and independence. Thus, we believe that their family interaction may present unique characteristics[22].

The purpose of this study was to explore the characteristics of family interaction among Chinese YBCSs, which could aid to the tailored interventions to improve their quality of life. The overall research question guiding this study was as follows: What are the characteristics of the interaction pattern between YBCSs and their family members in the Chinese family environment?

## Methods

### Design

We used semi-structured interviews, basic qualitative descriptions [23] and a content analysis to understand the characteristics of family interaction from the perspectives of YBCSs in China. Qualitative descriptions aim to provide a comprehensive summary of events in terms of the participants’ experiences and perceptions [23]. A content analysis is used to examine face-to-face human interaction. We believe that a content analysis is an ideal method. A content analysis requires the theoretical framework or theory to be identified before the data analysis begins [24]. Because the study’s view originates from Chinese female breast cancer survivors and patriarchy is a typical cultural norm in China [25], we chose Juliet Mitchell’s conceptualization of feminism to guide the YBCSs’ interactive behaviours. Mitchell’s conceptualization of feminism compromises the following 4 key structures: production, reproduction, sexuality, and the socialization of children [26]. Furthermore, social support represents an important interaction with family members, and we chose Langford’s conceptualization of social support (motional, instrumental, informational, and appraisal support) to guide the analysis of supportive interactions [27]. We report our study design and findings according to the Consolidated Criteria for Reporting Qualitative Research (COREQ) checklist.

### Participants

Purposive and snowballing sampling were used to recruit the participants (*n* = 17) between May 4, 2019 and January 8, 2020 [28]. Fourteen participants were recruited from the ward offices of two general hospitals in Shanghai, and 3 participants were referred by other participants. All potential participants were informed of the aims, objectives and methods of the study. The inclusion criteria for the participants were as follows: (a) Chinese women who (b) were aged 18 to 45 years, (c) had received a stage I-III breast cancer diagnosis, (d) had 6 months to 5 years survivorship from diagnosis, (d) had no prior history of breast cancer, and (e) were able to read and speak Chinese. The participants were asked semi-structured questions related to their experience after the cancer diagnosis; changes in their individual, interpersonal, community, and social environments; and the facilities and barriers to their recoveries.

### Ethical considerations

This study was approved by the Institutional Review Board of the institution where the researchers were affiliated. Furthermore, we obtained verbal and written informed consent from each participant before their participation.

### Data collection procedures

An experienced qualitative researcher interviewed 11 YBCSs in hospitals and 6 YBCSs via WeChat, the most popular social media platform in China. WeChat’s core function, video chatting, was used for the interviews. First, the researcher asked about the participants’ demographic information, including their age, education, marital status, religion, stage of cancer, type of treatment, and time of diagnosis, at the beginning of the interview. Then, a semi-structured interview guide was used to ask questions related to the characteristics of family interaction among YBCSs. The interview guidelines were as follows:

- Could you tell me your experience after you were diagnosed with cancer?
- What was the change in your interpersonal environment?
- How about your interaction with your family members?
- How about your interaction with others?
- Is there anything else you would like to tell me?

The interviewer encouraged the participants to express their genuine feelings by actively listening without interruption or judgement. All interviews were recorded and transcribed verbatim in Mandarin. Then, each respondent’s transcript was emailed to her to ensure that the expressions and statements were accurate. The transcripts were supplemented and modified according to the feedback provided by each participant.

### Data analysis

A content analysis was performed for the data analysis. The coding scheme was based on Mitchell’s four key elements: production (women’s presence in the work force), reproduction of children (women’s capacity for motherhood), sexuality (women’s sexual experience), and the socialization of children (women’s biological ‘destiny’ as a mother) [26]. These elements provided the basis for analysing the roles of women as the basis of interaction between YBCSs and their family members. Emotional (a subjective feeling of belonging, being accepted, being loved, and being needed), instrumental (provision of tangible goods and services or tangible aid, such as giving financial assistance or performing assigned work for others), informational (information provided to another during a time of stress), and appraisal support (affirmational support) provided an analytical perspective of the interactions between family members and YBCSs [27].

Each transcript was coded and analysed using the above frameworks as initial codes. An analysis team comprising an experienced qualitative researcher and a nurse, both with backgrounds in breast cancer and qualitative research, was formed to assist with the interpretation of the text. First, the data were open coded using a line-by-line coding process. The two analysts engaged in data immersion to become completely familiar with the data. A letter, word, sentence, or portion of the page was coded as the unit of analysis. The analysts carefully read the transcripts to identify evidence of the theory-derived codes. Repetitive words, concepts and phrases were recognized. Then, the codes were grouped into broader categories reflecting the characteristics of interactions between YBCSs and family members. Finally, the coding and categories of each transcript was compared between the analysts, and discrepancies were discussed and resolved to ensure consistency by the research team [29]. The analysis ceased when no new information emerged from the interviews (data saturation).

### Rigour

The scientific rigour of the study was achieved as follows: the research members clearly understand the design of the study, especially the role of the two theories, which is a foundational issue in content analysis. A coding scheme developed under the guidelines of theories and discussion after an independent analysis were used to increase the validity of this study. An experienced qualitative expert supervised the study process to ensure the reliability of the study [29].

## Results

**Fig. 1:**
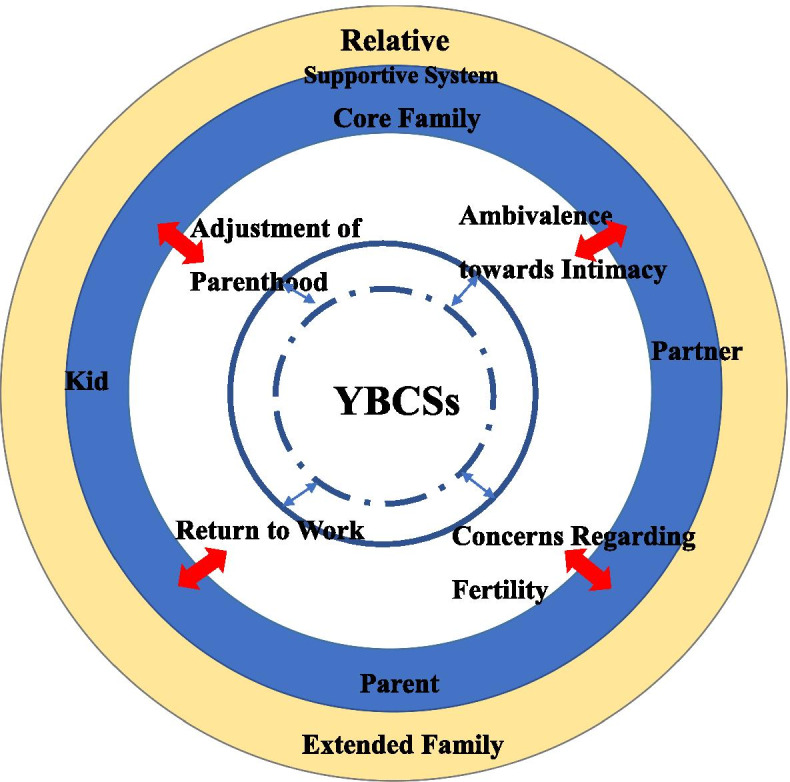
Family interaction among young Chinese breast cancer survivors

Seventeen YBCSs were recruited in this study (Table 1). We propose the characteristics of family interaction among Chinese YBCSs using a family interaction model based on our study findings (Fig. 1). Family interaction was categorized into the following 5 themes: adjustment of parenthood, ambivalence towards intimacy, concerns regarding fertility, return to work, and activation of the support system in a large family. The Chinese family is a large symbiotic system that includes parents, partners, children, and relatives in the extended family. Family members provide economic, emotional, and physical support to each other to achieve balance and development in the whole family. YBCSs have a fixed and internal capacity to adjust their behaviours towards family interaction and show personal resilience. When a cancer diagnosis disrupts the symbiotic balance, the role function of the female family member who is diagnosed will decrease or is even lost. Therefore, YBCSs attempt to adjust their interaction with family members to achieve a new balance, and family members respond by increasing their functional scope to cope with the cancer trauma experience.

**Table 1:** Demographic data of young breast cancer survivors (*N* = 17)

Number	Age	Disease stage	Child	Survivorship (years)	Education	Employment at interview	Marital status	Religion
N1	33	III	1	1	High school	Unemployed	Married	No
N2	45	II	1	1	University	Unemployed	Unmarried	No
N3	35	II	1	1	High school	Housewife	Married	No
N4	40	III	0	1	University	Unemployed	Unmarried	Buddhism
N5	43	II	1	1	High school	Unemployed	Married	No
N6	32	I	1	3	University	Part-time job	Married	No
N7	34	II	1	2	University	Unemployed	Married	No
N8	37	I	1	5	University	Housewife	Married	No
N9	38	II	1	2	High school	Employed	Married	No
N10	43	II	1	2	High school	Employed	Married	No
N11	40	II	2	2	University	Housewife	Married	No
N12	34	III	1	1	University	Unemployed	Married	No
N13	25	II	0	1	University	Part-time job	Unmarried	No
N14	29	I	0	1	University	Student	Unmarried	No
N15	28	II	0	1	University	Employed	Unmarried	No
N16	24	II	0	1	University	Employed	Unmarried	No
N17	37	I	1	2	University	Unemployed	Married	No

### Adjustment of parenthood

Children play an important role in the quality of life of many young cancer survivors. YBCSs are likely to have school-aged children living at home. The YBCSs included in this study attempted to adjust their parenting practices by changing their child-rearing approaches and engaging in effective communication to help the family adapt to the cancer crisis.

#### Changes in child-rearing approaches

Many young Chinese mothers attach great importance to their children’s education and push their children to receive high marks. Cancer forced the YBCSs to reconsider the value of health and the meaning of a good life that they could pursue. Many participants attempted to change their mental status because they considered an aggressive attitude and negative emotion to be the causes of the disease. In addition, the disease decreased their abilities to arrange everything for their children. Therefore, the YBCSs shifted their attention to their children’s physical and mental health rather than “forcing” their education. The participants developed a mutual respect style for communication and achieved cohesive family relationships.

No. 11 stated the following:

“In the past, I paid great attention to her (the survivor’s daughter’s) studies. I take her study seriously. Now, I don’t value scores greatly. Both of us feel much easier than before.”

Another mother with a boy in high school (No. 9) said the following:

“In the past, he (the son) yelled at me if I asked him to study. Now, I say nothing. I sit next to him and read a book by myself. Gradually, he puts down the cell phone and studies too*.*”

#### Perception of children’s care

The YBCSs perceived caring and support from their children. Such emotional support motivated them and gave them strength to persist with their oncological therapy.

A mother with a child in preschool (No. 7) said the following:

“My son came over and told me, ‘Mom, take a good rest. You must firmly believe that your illness can be cured.’ He gave me much confidence. I must live well and see his marriage and children.”

### Ambivalence towards intimacy

The YBCSs struggled to have close relationships with their partners. The participants desired to get married or maintain a marriage. However, many YBCSs with low self-esteem felt stressed due to intimacy, which created a contradictory status related to intimacy.

#### Desire for intimate relationships

The single YBCSs maintained aspirations for love and marriage. The married participants also desired a close relationship with their partners.

No. 14 said the following:

“I am young. I wish I could still meet the right man. However, I do not want to make it [an intimate relationship] necessary. Let it be.”

#### Perceive relationship insecurity

The YBCSs expressed an uncertain feeling regarding their marital status and aimed to avoid sexual activity due to the internalized stigma of cancer.

No. 11 said the following:

“My sex life is totally different from before. After all, I have had an operation on my body, which made me embarrassed. It (the cancer) is a shadow for both of us. To be honest, I have an inferior sex life.”

No. 14 said the following:

“I was still in chemotherapy treatment. He (my boyfriend) proposed we break up. This was a huge blow to me**.** I took a long time to recover. It is really hard for me.”

### Concerns regarding fertility

Fertility was a common concern among the YBCSs, especially among the childless survivors. The YBCSs maintained the hope of childbearing. However, the impact of adjuvant breast cancer therapy and the heritability of cancer reduced their potential eligibility for fertility preservation.

One survivor who had a baby after 3 years of treatment (No. 6) recalled the following:

“You should wait for several years to have a child. You will worry about the health of the baby because you are at an advanced maternal age. Will the chemotherapy lead to any side effects? You will definitely worry about it. It is a serious problem.”

No. 15 said the following:

“My grandfather was diagnosed with lung cancer. My mother passed away from breast cancer. Now, I have the same disease. I dare not have a kid. I am the third generation in my family to have cancer. How could I bring a fourth generation to this cancer family?”

### Return to work

Almost all survivors expressed both gratitude and guilt towards their family members for their support and cancer-induced burden. The participants also confirmed their willingness to return to work to resume their function as a core member of the family.

#### Coping with gratitude and guilt by working

The YBCSs showed gratitude for the support of all their family members. However, they struggled with guilt for causing their families stress and placing the disease burden on them. To reduce their guilt, they remained positive and exhibited a willingness to work with the people around them, especially YBCSs from one-child families.

No. 16 recalled the following:

“I know a sister, who is about 30 years old. She works harder after the diagnosis. She probably worried her parents greatly. She spent much money during the treatment. She is the only child in the family. She thought a lot for her ageing parents.”

The participants seldom communicated with family members about their negative emotions, such as worry or nervousness, and various health issues.

No. 15 said the following:

“Sometimes, I dare not talk to the people around me. In fact, they are worried, too. If I tell them my issues, it seems that I add a burden to them. I am very afraid of my negative emotion to have a bad impact. Therefore, I present myself as I wish to be seen, and I encourage them sometimes.”

#### Readapting to family and society by working

The YBCSs adjusted and found opportunities to work again. They rebalanced family with work and resumed their role functions in the family and society. Most YBCSs were stressed but still had to fulfil their role as a female (e.g., wife, mother, and daughter) in the family and a producer in society. Fulfilling these roles was an important way to achieve self-worth for the YBCSs, who still had long lives ahead of them.

No. 8, who had a daughter, said the following:

“As a woman, if you stay at home, you have to rely on your husband because you have no income. You would feel bored. If you work, you have to deal with many things related to the kids, such as teaching homework, attending parent meetings, etc. You cannot ask for leave from the company frequently. And you have to make some achievements. Otherwise, you will be fired by the company or replaced by a young man. So, females suffer great pressure from family and work. It is unfair, but no, you have to.”

### Activation of the support system in a large family

For YBCSs with large families, the family support system was activated in response to the cancer crisis. However, Chinese family members seldom express their love and support in words. They usually provide support through their actions, such as by providing care and silent accompaniment.

#### Instrumental support from core family members

The core family system, including parents, partners and children, provided instrumental support (physical, financial, and emotional support and therapeutic decision making). They provided support, such as financial assistance with paying hospital bills, routine physical care, and diet management. No. 8 recalled the following:

“The family is very important in knowing how to support the patients. You need to know how to manage the daily food of the survivor in addition to the economic strategy. How long is the period of each treatment? How to cooperate with the physician for treatment?”

However, the emotional support offered by family members was more implicit, and the most common form of such support was simply “***being there***”. No. 16 said the following:

“I know that they (family members) support me and love me greatly. I know they will never give up on me, even though we have little communication.”

#### Support from relatives

Relatives assisted the YBCSs and their core family members through instrumental, informational and appraisal support. Information support included providing health treatment and diet information. Sharing the positive stories of other YBCSs was a form of appraisal support reported by the participants. The other significant form of instrumental support from relatives was to provide help caring for children during the treatment; for instance, No. 3, who had a 7-year-old daughter, said the following:

“My husband’s sister took care of my daughter. My daughter often has dinner with them. Then, my husband will take her home after he finishes his work.”

## Discussion

### Overview of the findings

The aim of this paper was to explore how Chinese YBCSs interacted with their family members to cope with and adapt to a cancer crisis. In this exploration, we used Juliet Mitchell’s key structures to understand the role functions of women and interpret the pattern of family interaction among YBCSs [26]. Our findings support and enrich the content of Mitchell’s theory. As proposed, the combination of production, reproduction, sexuality and the socialization of children describes the “complex unity” of women’s position and manifests in their interactive performance [26]. In the population of Chinese YBCSs, these four areas are influenced by the unique Chinese culture. Our study also shows that family members engaged in supportive behaviours to increase family resilience through social support [27].

All human interactions and family patterns are influenced by the social and cultural settings in which they occur [30–32]. Thus, Chinese social-cultural factors allowed us to obtain an in-depth understanding of YBCSs’ family interaction in this study. Family is the centre of most Chinese women’s lives [33]. Influenced by Confucian philosophy, Chinese parents place a high value on education [34]. Tiger mothers with high academic expectations are very common in Chinese families [35, 36]. These mothers are authoritarian and even restrict their children’s extracurricular activities, leading to a negative effect due to their intense relationships with their children [36, 37]. In the broader context, there is an emphasis on supportive parenting, which is another cultural dimension influencing the attitudes of young mothers according to Rogoff [32, 38]. In this sense, cancer becomes a point for reflection upon parenthood. The perception of breast cancer as threatening and stressful is an important facilitator of posttraumatic growth for breast cancer survivors, especially YBCSs [39]. The participants changed their child-rearing style to emphasize mutual respect and an understanding relationship. We believe that the adjustment of parenthood was the product of a complicated influence of traditional values, self-reflection, and physical functions.

The participants showed conflicting attitudes towards intimacy with their partners and fertility issues. Reproductive problems and sexual dysfunction are common issues among YBCSs across various countries [40–42]. Chinese YBCSs also desire intimate relationships. Married YBCSs hope to maintain sexual activity. It is well known that throughout history, women have been appropriated as sexual objects, especially in ancient China, and males exhibit extraordinary dominance in families [26]. However, treatment sequelae, including biological factors (such as pain during sex, vaginal dryness, and fatigue) and psychological factors (such as low body image and depression), lead to sexual inactivity [41]. Additionally, Asian women are expected to be self-sacrificial and avoid causing trouble for others [33, 43]. Self-blame and avoidance are coping strategies that Asian women usually adopt. These women want, but do not know how, to discuss such problems with their partners, leading to negative dyadic coping and a poorer sexual relationship [43]. Unmarried survivors maintain the hope for a romantic relationship but do so passively, especially those who experienced breakups after their diagnosis. Fertility preservation is another common need for single survivors and survivors without children [41, 42]. Understandably, survivors with children are not very concerned about fertility issues since they have no strong wish for more children due to the long-standing influence of the one-child policy. This situation differs from that of cancer survivors in other countries [41, 44].

The YBCSs seldom communicated health issues and their negative emotions to their family members, especially their parents. Chinese people emphasize that younger people should care for elderly people to show their filial piety; thus, a YBCS may feel that if she does not care for her elderly parents, she is not a good daughter. Since most young survivors are from the one-child generation in their families, we understand that they suffer a high level of stress. Generally, they would like to show positivity in front of their family members as a form of support. They seek emotional and experiential support from their peers with cancer through the Internet or physicians [44].

The Chinese YBCSs in this study perceived great instrumental, informational, and appraisal support from their parents, partners, and siblings, which is consistent with Pamela’s study involving African YBCSs [42]. However, emotional support from the YBCSs’ family members was implicit and manifested in the feeling of “being there with you”. Family members showed their love through physical caring and accompaniment, which could be explained by the concept of high-context cultures proposed by Edward Hall [45]. In contrast to Western low-context cultures, where survivors seek support through explicit words, such as encouragement, holding hands, and hugging [44], living in a typical high-context culture, Chinese YBCSs tend to seek supportive signals through body language and the use of silence.

### Strengths and limitations

One strength of this study is the active exploration of Chinese YBCSs’ family interaction patterns. This study enriches our understanding of young females with breast cancer, who constitute an underserved population.

We examined the interaction patterns in YBCSs’ families by analysing the survivors’ perceptions of and experiences with their family members. However, the family members’ perceptions and experiences are missing and cannot be used to verify and enrich the interaction patterns. Furthermore, it is important to explore the family-society interaction patterns from an integrated micro–macro perspective. We did not investigate survivors and their family members in various areas and ethnic groups from rural and urban areas, which are characterized by different cultural and social environments. In the future, we could also divide survivors into detailed age groups as survivors of different ages have different personal goals and coping capacities according to the position and role functions of females expected by society. Finally, we used WeChat to conduct some interviews. Interviewing via WeChat Video is convenient and flexible for participants and researchers and is not subject to time and space limitations. It is easier for participants to express their perceptions when the interaction is on screen rather than face-to-face. However, a disadvantage of online interviews is that it is difficult for the interviewer to observe all reactions, especially body gestures.

## Conclusions

This study contributes to a deeper understanding of the interactions between YBCSs and their family members in China. The Chinese YBCSs and their families developed typical interactive patterns to cope with the cancer trauma and achieved a new form of a family interactive pattern. Health professionals could develop female-sensitive, culturally specific interventions for Chinese YBCSs and their families to support positive interactions and increase family resilience as well as quality of life.

## Supplementary Information

**Additional file 1.**

## Data Availability

The datasets generated and/or analysed during the current study are available from the corresponding author upon reasonable request.

## Abbreviation

YBCSs: Young breast cancer survivors
