# Supplementary material for: Family interaction among young Chinese breast cancer survivors
Source: BMC Fam Pract. 2021 Jun 21;22:122. doi: 10.1186/s12875-021-01476-y (PMC8218435; doi:10.1186/s12875-021-01476-y)
Supplement: Supplementary file 1 — Additional file 1. [file 12875_2021_1476_MOESM1_ESM.doc]

**The detailed interview guidelines**

(a) Could you tell me your experience after you were diagnosed with cancer?

(b) What was the change in your interpersonal environment?

- What are the facilitators for the change? And barriers?

(c) How about your interaction with your family members?

- What do you think about work?
- What do you think about the fertility issue?
- How about your sexual life?
- What do you think about the relationship with your children?
- What support could get from your family members? Who?

(d) How about your interaction with others?

(e) Is there anything else you would like to tell me?
